# Improvement of CO_2_ and Acetate Coupling into Lactic Acid by Genetic Manipulation of the Hyperthermophilic Bacterium *Thermotoga neapolitana*

**DOI:** 10.3390/microorganisms9081688

**Published:** 2021-08-09

**Authors:** Nunzia Esercizio, Mariamichela Lanzilli, Marco Vastano, Zhaohui Xu, Simone Landi, Lucio Caso, Carmela Gallo, Genoveffa Nuzzo, Emiliano Manzo, Angelo Fontana, Giuliana d’Ippolito

**Affiliations:** 1 Institute of Biomolecular Chemistry (ICB), Consiglio Nazionale delle Ricerche (CNR), Via Campi Flegrei 34, 80078 Pozzuoli, Italy; n.esercizio@icb.cnr.it (N.E.); m.lanzilli@icb.cnr.it (M.L.); marco.vastano@gmail.com (M.V.); l.caso@icb.cnr.it (L.C.); carmen.gallo@icb.cnr.it (C.G.); nuzzo.genoveffa@icb.cnr.it (G.N.); emanzo@icb.cnr.it (E.M.); afontana@icb.cnr.it (A.F.); 2 Department of Biological Sciences, Bowling Green State University, Bowling Green, OH 43403, USA; zxu@bgsu.edu; 3 Laboratory of Bio-Organic Chemistry and Chemical Biology, Department of Biology, University of Naples “Federico II”, Via Cinthia, 80126 Napoli, Italy; simone.landi@unina.it

**Keywords:** L-lactic acid, hydrogen, acetate assimilation, CO_2_ absorption, hyperthermophile engineering

## Abstract

Capnophilic lactic fermentation (CLF) represents an attractive biotechnological process for biohydrogen production and synthesis of L-lactic acid from acetate and CO_2_. The present study focuses on a genetic manipulation approach of the *Thermotoga neapolitana* DSM33003 strain to enhance lactic acid synthesis by the heterologous expression of a thermostable acetyl-CoA synthetase that catalyses the irreversible acetate assimilation. Because of the scarcity of available genetic tools, each transformation step was optimized for *T. neapolitana* DSM33003 to cope with the specific needs of the host strain. Batch fermentations with and without an external source of acetate revealed a strongly increased lactate production (up to 2.5 g/L) for the recombinant strain compared to wild type. In the engineered bacterium, the assimilation of CO_2_ into lactic acid was increased 1.7 times but the hydrogen yield was impaired in comparison to the wild type strain. Analysis of fermentation yields revealed an impaired metabolism of hydrogen in the recombinant strain that should be addressed in future studies. These results offer an important prospective for the development of a sustainable approach that combines carbon capture, energy production from renewable source, and the synthesis of high value-added products, which will be addressed in future studies.

## 1. Introduction

Thermophilic and hyperthermophilic bacteria represent attractive and still poorly explored candidates in designing efficient microbial cell factories for target bioprocesses. High temperature biotransformation, in fact, provides numerous technological advantages, including (i) reduced contamination risks, (ii) increased solubility of renewable substrates such as lignocellulosic biomasses, (iii) continuous recovery of volatile chemical products directly from reactor headspace, and (iv) decreased cooling costs [1,2]. Moreover, for biohydrogen production, the process becomes thermodynamically more favourable at high temperatures, thus increasing the overall productivity [3].

Among hyperthermophilic bacteria (over 80 °C), microorganisms belonging to the order *Thermotogales* have the ability to convert several types of carbohydrate-rich biomasses into H_2_ by dark fermentation (DF) with yields close to the Thauer limit of 4 moles of hydrogen per mole of glucose, according to the following reaction [4,5,6,7,8,9,10]:

C_6_H_12_O_6_ + H_2_O → 2 CH_3_COO^-^ + 2 CO_2_ + 2H^+^ + 4 H_2_ (1)

The ΔG° of this reaction is −206.3 kJ mol^−1^ [4]. Few species can approach this limit due to thermodynamic limitations and metabolic requirements such as maintaining a supply of NADH that can be met by lactate production.

**Figure 1:**
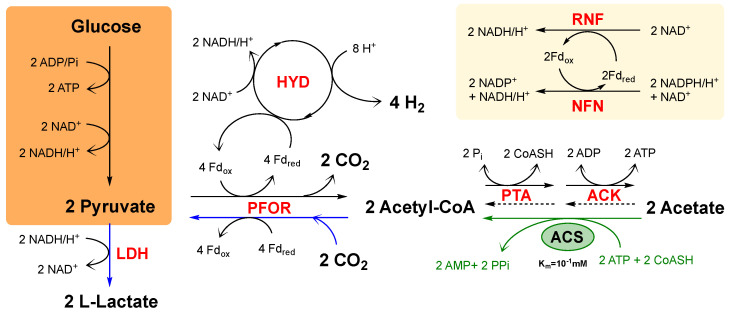
Schematic representation of the biochemical pathway underling the CLF process. Plain black arrows indicate the catabolic branch; blue arrows indicate the anabolic branch; green arrow indicates the ACS heterologous insertion. Boxes indicate glycolysis (orange) and putative NADH production by flavin-based oxido-reductase enzymes (pale yellow). ACK, acetate kinase; PTA, phosphotransacetylase; ACS, acetyl-CoA synthetase; PFOR, pyruvate:ferredoxin oxidoreductase; LDH, lactate dehydrogenase; HYD, hydrogenase; RNF, NAD-ferredoxin oxidoreductase; NFN, NADH-dependent reduced ferredoxin:NADP oxidoreductase; Fd, ferredoxin. Green arrow indicates the ACS heterologous insertion.

*Thermotoga neapolitana* is a rod-shaped bacterium of approximately 1.0–10.0 μm in length and 0.4–1.0 µm in diameter. The bacteria are wrapped by an external membrane named “toga” [5,6] that forms an outer sheath ballooning over the ends [11]. A few years ago, we reported a novel anaplerotic process, named capnophilic lactic fermentation (CLF) (capnophilic means “requiring CO_2_”), that enables a non-competitive synthesis of L-lactic acid (LA) and hydrogen in *T. neapolitana* [12,13,14,15]. As shown in Figure 1, the fermentation process is activated by CO_2_ and, nominally, is dependent on a Janus pathway, which includes a catabolic branch leading to acetyl-CoA (Ac-CoA) from sugars by glycolysis as well as an anabolic branch converting Ac-CoA and CO_2_ to pyruvate by PFOR (pyruvate:ferredoxin oxidoreductase; EC 1.2.7.1) and synthesizing LA by lactate dehydrogenase (LDH; EC 1.1.1.27) [12,14,15,16,17]. In addition to energetic flow derived from glycolysis, analysis of the transcripts suggested that flavin-based oxido-reductase enzymes such as NADH-dependent reduced ferredoxin: NADP oxidoreductase (NFN) and NAD ferredoxin oxidoreductase (RNF) supply reduced ferredoxin and NADH to support concomitant synthesis of lactic acid and hydrogen [17].

In *T. neapolitana*, the acetate (AA) dissimilation pathway is supported by the reversible enzyme phosphate acetyltransferase (PTA; EC 2.3.1.8) and acetate kinase (ACK; EC 2.7.2.1). PTA converts acetyl-CoA and inorganic phosphate to acetyl-P and CoASH, while ACK converts acetyl-P and ADP to acetate and ATP [18]. AMP-AcetylCoA synthetase (ACS; EC 6.2.1.1) is considered a key enzyme involved in the irreversible acetate assimilation but not found in the *T. neapolitana* genome. In contrast to the ACK-PTA system, ACS first converts acetate and ATP to the enzyme-bound intermediate acetyladenylate (acetyl-AMP) while producing pyrophosphate. It then reacts acetyl-AMP with CoASH to form acetyl-CoA, releasing AMP [19,20]. Metabolic engineering targeting the intracellular acetyl-CoA pool is typically conducted to enhance the acetyl-CoA supply for producing high-value added chemicals using acetyl-CoA as a precursor [21]. Heterologous expression of ACS has been described as a good strategy to increase acetate assimilation pathway [22].

The aim of the present study was to boost the flow from CO_2_ and acetyl-CoA to LA (Figure 1—blue arrows) in order to increase the fixation rate of environmental CO_2_ into LA and overtake the production of CO_2_ from glycolysis (Figure 1—black arrows). Considering the upregulation of PFOR and LDH under CO_2_ [17], an increase of the upstream acetate uptake could improve the acetate and CO_2_ coupling. Although disruption of ACK and/or PTA may represent a valid approach to increase acetyl-CoA availability, methodology for knock-out of specific gene in *T. neapolitana* has not been reported. The strategy of this work was to increase acetate uptake in *T. neapolitana* by heterologous expressing a *Thermus thermophilus* ACS (Figure 1—green arrow). To achieve this, we adopted genetic manipulation techniques that were developed for other *Thermotoga* strains and optimized them for *T. neapolitana* DSM 33003, which is a strain adapted to saturating CO_2_ concentration [23].

## 2. Materials and Methods

### 2.1. Strains and Growth Conditions

*Thermotoga neapolitana* subsp. *capnolactica* (DSM 33003) derives from the DSMZ 4359T strain that was isolated in our laboratory under saturating concentrations of CO_2_ [23]. Bacterial cells were grown in a modified ATCC 1977 culture medium containing 10 mL/L of filter-sterilized vitamins and trace element solution (DSM medium 141) together with 10 g/L NaCl, 0.1 g/L KCl, 0.2 g/L MgCl_2_ × 6H_2_O, 1 g/L NH_4_Cl, 0.3 g/L K_2_HPO_4_, 0.3 g/L KH_2_PO_4_, 0.1 g/L CaCl_2_ × 2H_2_O, 1 g/L cysteine–HCl, 2 g/L yeast extract, 2 g/L tryptone, 5 g/L glucose, and 0.001 g/L resazurin [17]. Resazurin was used as redox-sensitive indicator of oxygen levels in the medium. Media was aliquoted in 120 mL serum bottles, with 30 mL of culture. Anaerobic conditions were obtained by heating the medium until the solution became colourless. Serum bottles were sealed, capped, and autoclaved for 10 min at 110 °C. When needed, 2-^13^C-sodium acetate 20 mM was added into medium from a 60× stock solution, after filtration at 0.22 µm. Kanamycin and chloramphenicol were tested at 80 °C at different concentrations to test strain sensitivity (kanamycin: 200, 350, 400, and 500 µg/mL; chloramphenicol: 30, 50, 100, 150, and 300 µg/mL). Chloramphenicol at 200 µg/mL was used for selection of transformed strains. For routine experiment, bacterial precultures were incubated overnight at 80 °C without shaking and used to inoculate (6% *v*/*v*) cultures in 120 mL serum bottles with a final culture volume of 30 mL. Cultures were sparged with CO_2_ gas for 5 min at 30 mL/min. Inoculated bottles were maintained in a heater (Binder ED720) at 80 °C. Every 24 h, 2 mL of samples were collected from each bottle, using a syringe through the rubber septum without altering the anaerobic atmosphere. The aliquots were centrifuged at 16,000× *g* for 15 min (Hermle Z3236K, Wehingen, Germany), and supernatants were stored at −20 °C until analyses. Every 24 h, pH was monitored, CO_2_ was sparged for 5 min, and pH was adjusted to approximately 7.5 by 1 M NaOH. Cell growth was determined by measuring optical density (OD) at 540 nm (UV/Vis Spectrophotometer DU 730, Beckman Coulter, Pasadena, CA, USA). Cell morphology and spheroplast formation were monitored by microscope observation (Axio VertA1, Carl Zeiss, Oberkochen, Germany, magnification of 100×).

### 2.2. Gas and Chemical Analysis

H_2_ measurements were performed by gas chromatography (GC) (Focus GC, Thermo Scientific, Waltham, MA, USA) equipped with a thermoconductivity detector (TCD) and fitted with a 3 m molecular sieve column (Hayesep Q). N_2_ was used as the carrier gas. Glucose concentration was determined by the dinitrosalicylic acid method calibrated on a standard solution of 1 g/L glucose (Bernfeld, 1995). Organic acids were measured by ERETIC ^1^H NMR as described by Nuzzo et al. (2019). All experiments were performed on a Bruker DRX 600 spectrometer equipped with an inverse TCI CryoProbe. Peak integration, ERETIC measurements, and spectrum calibration were obtained by the specific subroutines of Bruker Top-Spin 3.1 program. Spectra were acquired with the following parameters: flip angle = 90°, recycle delay = 20 s, SW = 3000 Hz, SI = 16K, NS = 16, and RG = 1. An exponential multiplication (EM) function was applied to the FID for line broadening of 1 Hz. No baseline correction was used. For carbon balance, CO_2_ was estimated according to the amount of produced acetic acid. For the correlation between cells dry weight (CDW) measurement and OD_540nm_, a previously determined equation was used: CDW (g/L) = 0.347 × OD_540nm_ + 0.016 [24]. The chemical composition of the dry cells was estimated adapting the empirical formula C_5_H_8_O_3_NS_0.05_ reported in literature [25]. Data were averages of eight biological replicates for culture without and three biological replicates for culture with exogenous acetate.

### 2.3. Construction of Vectors

Vectors were constructed by following standard cloning methods and verified by restrictive digestions. The *Thermotoga*-*E. coli* shuttle vector pDH10 was used as the parent vector. DNA sequences were synthetized by GeneArt™, after codon usage optimization (Figure S1). Our selective marker was the chloramphenicol acetyltransferase (*cat*) gene variant A138T from *Staphylococcus aureus*, which was evolved by Kobayashi et al. (2015) for higher thermal stability [26]. The *cat* gene was cloned between EcoNI and EcoRI in place of the *kan* gene in pDH10, resulting in plasmid pGD11. The *acs* gene from *Thermus thermophilus* H8 (TTHA1248) was inserted downstream of the promoter sequence of *T. thermophilus* H8, which was already reported by Han et al. (2012) to be active in *Thermotoga* and was cloned into XbaI-SacI pGD11 to give rise to pGD11-ACS [27].

### 2.4. Spheroplast Formation and Transformation

Preparation of spheroplasts was adapted from a protocol already reported for *T. neapolitana* [28]. Briefly, 50 mL of *T. neapolitana* cells in early stationary phase was harvested and washed twice with WB solution [300 mM KCl, 2 mM MgSO_4_ and 40 mM K_2_HPO_4_ (pH 7.0). After centrifugation at 16,000× *g* for 15 min, spheroplasts were prepared by resuspending the pelleted cells in 500 µL of WB containing 350 mM sucrose, 2 mg/mL EDTA and 2 mg/mL lysozyme. The cell suspension was incubated at 37 °C and spheroplast formation was monitored by optical microscope at magnification 100× and reticulated lens. Efficiency of toga removal was estimated by the ratio between round cell number and total cell number. After 90 min of treatment, an efficiency of 80% in spheroplast formation was estimated. The reaction was stopped by incubation at 77 °C for 5 min. Spheroplasts were centrifuged and resuspended in the appropriate buffer to test different transformation protocols (natural transformation, liposome-mediated transformation, and electroporation-mediated transformation). For natural transformation, as reported by Han et al. (2014), 1 mL of the overnight culture or spheroplast preparation, obtained as described above, was collected by centrifugation, resuspended in 200 μL of fresh medium, and was injected into a 100 mL serum bottle containing 10 mL of fresh medium; DNA substrate was added to a final concentration of 5 μg/mL [29]. Then, the culture-DNA mixture was incubated at 80 °C for 4 to 6 h with gentle agitation (100 rpm) and then transferred into 100 mL serum bottles containing fresh medium, with a supplement of 200 µg/mL chloramphenicol for selection. Growth was monitored for 48 h. For liposome-mediated transformation, as reported by Yu et al. (2001), spheroplasts were resuspend in 1 mL solution (pH 7.4) of 4.5 mM NH_4_Cl, 0.3 mM CaCl_2_, 0.34 mM K_2_HPO_4_, 22 mM KCl, 2 mM MgSO_4_, 340 mM NaCl, and 20 mM HEPES [30]. A DNA: liposome mixture was prepared by mixing 5 µg DNA to 20 µg DOTAP in a total volume of 100 µL of 20 mM HEPES buffer (pH 7.4). After 15 min of incubation at room temperature, the mixture was added to the spheroplasts suspension and incubated for 1 h at 37 °C. A 0.5 mL portion of the spheroplasts suspension was transferred into 10 mL medium in a serum bottle and incubated at 77 °C for cell recovery (5 h) and then inoculated in medium containing the antibiotic. For electroporation-mediated transformation, spheroplasts were resuspended in 300 µL of electroporation buffer containing 10% glycerol and 0.85 M sucrose solution (EB), which was adapted from Han [27]. Two different amounts of spheroplast preparations (10^7^ and 10^8^) were tested for electroporation. Plasmid DNA (5 μg) was mixed with 300 μL of freshly made competent cells and incubated on ice for 5 min prior to introduction to a pre-chilled cuvette of 1 mm gap. For all operations, the capacitance was set at 25 μF, and the exponential pulses were tested with the following resistances and voltages: 400 Ω & 1.25 kV; 200 Ω & 1.8 kV; and 200 Ω & 2 kV (Gene PulserXcell™, Bio-Rad Laboratories, Hercules, CA, USA). After electroporation, 1 mL of fresh medium was added into each cuvette, and the cell suspension was transferred to 10 mL medium in a serum bottle and incubated at 77 °C with for 3 h for recovery and then inoculated in medium containing the antibiotic. Different amounts of the transformant cultures (2 mL, 5 mL, 10 mL, and 20 mL) were treated for plasmid isolation with QIAGEN miniprep kit according to manufacturer’s instructions. The same protocol was also applied to 5 mL culture of T. sp RQ7 strain, and cryptic plasmid pRQ7 was revealed, indicating that the extraction procedure was successful. For PCR analysis, KAPA Taq PCR kit (MERK) was used. For target amplification, following ACS primers were used: acs_Fw: CGCCAACGTGCTGAAAAGACTGGG; acs_Rev: GGCCAAGGTCTCGTGATACACAGG. PCR protocol was validated using pGD11-ACS extracted from *E. coli* as the template.

## 3. Results and Discussion

### 3.1. Assessment of Genetic Tools and Transformation Method

Genetic methods for the study of hyperthermophilic bacteria are at early stages of development. Although some examples of transformation of *Thermotogales* strains are reported [29,31,32,33], engineering approaches with these bacteria remain limited due to the technological barriers posed by their thermophilic and strictly anaerobic nature. Even fewer studies have been reported about the genetic transformation of bacteria belonging to *Thermotoga* genus (Table 1), and only the most recent reports focus on the development of auxotrophic strains [32,34] and knock-out of specific genes [33,35]. Therefore, considerable efforts have been paid to the development of methods to address an efficient transformation of DSM33003 (Figure S2). This work included identification of selection markers, preparation of competent cells, assessment of transformation techniques, and vector design.

In order to define a suitable selective marker, *T. neapolitana* DSM33003 was tested for its viability against kanamycin and chloramphenicol at 80 °C, which are thermostable antibiotics used for selection in *Thermotoga* species (Table 1). Due to the inconsistent results reported for the screening of transformed *T. neapolitana* on plate, the selection was carried out only in liquid media [30]. The thermostable antibiotics were tested at different concentrations ranging from 30 to 400 µg/mL by monitoring cell growth at 24 h, 48 h, and 72 h (Figure S3). Although kanamycin is the most widely used selective agent for bacteria of the genus *Thermotoga* (Table 1), DSM33003 is not sensitive to kanamycin, in agreement with another report on *T. neapolitana* [30]. On the contrary, chloramphenicol totally inhibited cell growth at concentration above 200 μg/mL and thus was chosen as the thermostable selective agent for DSM33003 at 200 μg/mL in liquid medium.

For vector design, the plasmid pDH10 (GenBank: JN813374) was used as the starting material. Vector pGD11 was constructed by replacing the Kan^r^ sequence of pDH10 with the evolved variant of chloramphenicol acetyltransferase (*cat*) from *Staphylococcus aureus* with increased thermal stability [26]. In addition to Kan^r^ sequence, pDH10 carries ColE1 origin of replication (*ori*) and ß-lactamase (Amp^r^) for amplification and selection in *E. coli*. Moreover, like pJY1, pDH10 relies on the sequence of the endogenous plasmid pRQ7 to guarantee replication in *Thermotoga* [30]. The heterologous sequence of ACS and the selected promoter from *T. thermophilus* HB8 were optimized according to codon usage of *T. neapolitana*, synthesized, and cloned into XbaI-SacI in pGD11, resulting in the plasmid pGD11-ACS (Figure S4).

It has been reported that the periplasmic space between toga and plasma membrane negatively affects the transformation of *Thermotoga* spp. as the wider the space correlates to the lower permeation of DNA [29]. In 2001, Yu et al. reported acquisition of transient chloramphenicol resistance in liquid culture of *T. neapolitana* after spheroplast formation [30]. Analogously, pDH10 vector has been used to confer kanamycin resistance to *T. maritima* and *T*. sp RQ7 by liposome-mediated transformation of spheroplast [27]. Removal of toga in *T. neapolitana* DSM33003 was obtained by adapting original protocol reported in Yu et al., 2001 [30]. Cells harvested in early stationary phase were treated with 3 mg/mL lysozyme and incubated for 90 min at 37 °C. This treatment induced formation of round spheroplasts clearly distinguishable from rod-shaped cells with toga by optical microscope at 100× (Figure S5).

### 3.2. Generation of Recombinant Strains

Among *Thermotoga* spp., DNA uptake has been achieved by electroporation, natural transformation, and liposome-mediated electroporation (Table 1). Various conditions were investigated, including different electroporation pulses (1.25 kW–400 ω, 1.8 kW–200 ω), amount of plasmid DNA (1 µg, 5 µg), and spheroplast concentration (10^7^, 10^8^). The most replicable condition assuring stable chloramphenicol-resistant *T. neapolitana* cells was electroporation at 1.8 kW−200 ω with 5 µg of pGD11-ACS. Natural transformation was successful only when applied to spheroplast suspension of the bacterial cells, indicating that this strain is not naturally competent [32].

**Figure 2:**
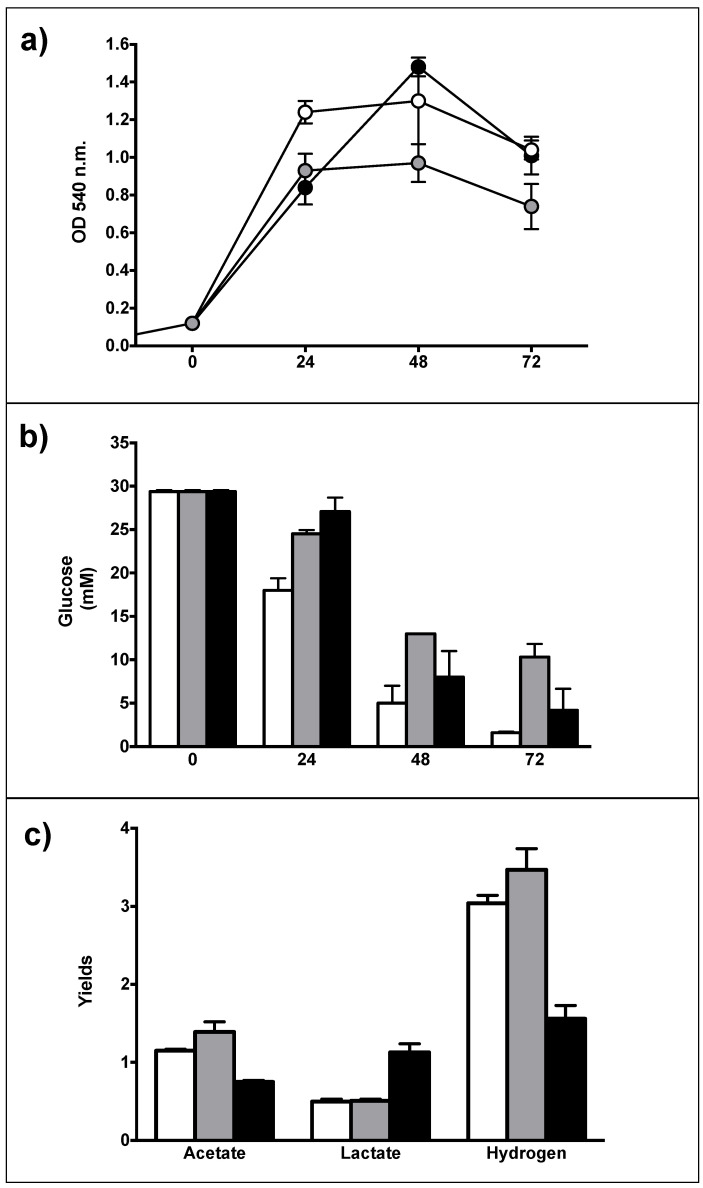
Batch fermentations of wild type (white circle/bars), empty vector (grey circle/bars) and ACS recombinant strain (black circle/bars) after 72 h in static condition. (**a**) Growth curves, expressed as OD_540nm_; (**b**) Glucose consumption, expressed as mM; (**c**) Fermentation products, expressed as yield (mmol product/mmol consumed glucose). Chloramphenicol was used at 200 µg/mL for selection of recombinant strains. Data are expressed as mean ± SD, *n* = 3.

Despite several attempts to reveal ACS sequence by PCR from the plasmid and genomic DNA preparations of *T. neapolitana* transformants, no amplification was observed (primer sequences in Figure S4). The absence of amplified ACS may be caused by low copy numbers of the shuttle vectors, although a chromosomal integration cannot be excluded at the moment. It is worth to note that for all episomial transformations reported for *Thermotogales,* the whole plasmid was never revealed, and only PCR amplified fragments were used as further proof of transformation in addition to resistance acquisition [27,29,30,31]. Phenotypical traits of recombinant strains represent an indirect way to demonstrate DNA perturbation. For example, Xu reported the absence of PCR amplicons of a heterologous cellulase gene in a recombinant *Thermotoga* sp RQ2 strain, which clearly expressed the expected extracellular cellulase activity [31]. Recombinant strains *T. neapolitana* DSM33003/pGD11-ACS (named *Acs03*) and *T. neapolitana* DSM33003/pGD11 (the empty vector) were characterized for their capacities to couple acetate and CO_2_ into LA in standard medium with antibiotic in comparison to the wild type strain (wt) in the standard condition without antibiotic (Figure 2).

Cells transformed with the empty vector showed a slowdown in growth compared to wt, probably due to a slight effect of the backbone plasmid including antibiotic resistance (Figure 2a). This influence was overcome in the *Acs03* recombinant strain, probably due to the occurrence of the heterologous gene or spontaneous mutation that improved the strain. In fact, *Acs03* accelerated growth and glucose consumption arising similar values of wt after 72 h of fermentation (Figure 2a).

Different yields of acetate (AA) and lactate (LA) were observed in *Acs03* compared to the empty vector and wt strains (Figure 2b). *Acs03* produced minor yield of AA (0.75 ± 0.02) in comparison to wt (1.15 ± 0.02) and empty vector control ((1.3 ± 0.13) and doubled LA yield from 0.5 ± 0.03 (wt and empty vector control) to 1.13 ± 0.11 mM (Figure 2b). Increase of lactate in *Acs03* is partially covered by decrease of acetate yield. Significantly, ratio LA/AA in *Acs03* was about three times higher than in wt (0.44) and strain with empy vector (0.36) (Table 2). H_2_ production was greatly impacted in *Acs03*, halving from 3.04 ± 0.1 mM to 1.56 ± 0.17 mM in *Acs03*.

### 3.3. Catabolic and Anabolic Origins of Lactate

**Figure 3:**
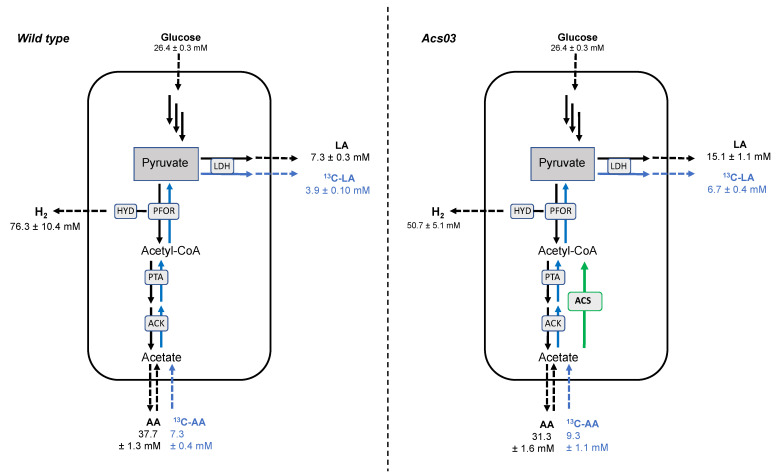
Capnophilic Lactic Fermentation (CLF) flow using 28 mM glucose and 20 mM 2-^13^C-acetate in the wild type and *Acs03* recombinant strain. Unlabelled lactate (LA) derived from catabolic pyruvate (glycolysis) and anabolic pyruvate produced by coupling reaction of CO_2_ and unlabelled acetate (AA) produced by fermentation. 2-^13^C-acetate (^13^C-AA) is internalized and metabolized to 3-^13^C-lactate (^13^C-LA). ACK, acetate kinase; PTA, phosphotransacetylase; ACS, acetylCoA synthetase; PFOR, pyruvate:ferredoxin oxidoreductase; LDH, lactate dehydrogenase; HYD, hydrogenase. Green arrow indicates ACS heterologous insertion. Data are expressed as mean ± SD, *n* = 3.

Generally, LA is formed from pyruvate reduction catalysed by LDH. Under CLF conditions, pyruvate can derive either from glycolysis (catabolic pyruvate) or enzymatic coupling of acetate and CO_2_ (anabolic pyruvate) (Figure 1). To assess the origin of increased level of LA in *Acs03*, the contribution of each pathway to LA production was measured with external labelled 2-^13^C-acetate [14]. Consumption of 20 mM exogenous 2-^13^C-AA and formation of 3-^13^C-lactate (3-^13^C-LA) were monitored in ^1^H-NMR spectra by integrating doublets flanking the methyl natural resonances of acetate at 1.9 ppm (methyl group of 2-^13^C-AA) and lactate at 1.33 ppm (methyl group of 3-^13^C-LA) (Figure S6). Although uptake of exogenous 2-^13^C-AA was quite similar for both strains (7.3 ± 0.4 mM for wt and 9.3 ± 1.1 mM for *Acs03*), capability to convert 2-^13^C-AA into 3-^13^C-LA was enhanced in the recombinant bacterium (Figure 3). Labelled LA was 1.7 fold higher in *Acs03* (6.67 ± 0.40 mM) in comparison to wt (3.90 ± 0.1 mM). Considering the stochiometric reaction, we assessed that *Acs03* recycle 293.5 ± 18 mg/L of CO_2_ and 547 ± 32 mg/L of acetate into 747 ± 45 mg/L of sodium lactate; in contrast, the wt recycled 172 ± 4.4 of CO_2_ and 320 ± 8.2 of acetate into 437 ± 11.2 mg/L of sodium lactate. Molecular reorganization induced by pGD11-ACS in the recombinant strain *Acs03* boosted the fixation of CO_2_ into lactate by 70%.

As stated above, unlabelled-LA can be derived from pyruvate produced by glycolysis and/or from AA uptake. During the fermentation, exogenous labelled AA is diluted with unlabelled-AA produced by glucose catabolism. Although flux quantification from 1-^13^C-AA into 3-^13^C-LA is an underestimation of total LA deriving by coupling reaction of CO_2_ and AA (endogenous + exogenous), it represents an unequivocal way to estimate the minimal flux, highlighting the improved performances of *AcS03* in comparison to wt.

Production of catabolic lactate identified as unlabelled-LA was also enhanced in the recombinant strain (from 7.3 ± 0.3 mM in wt to 15.1 ± 1.1 mM in *Acs03*), supporting a major flux from catabolic pyruvate to lactate. This should account for the reduction of hydrogen production from 76.3 ± 10.4 mM in wt to 50.7 ± 5.1 mM in *Acs03*. In dark fermentation, 2 mol of acetic acid and 4 mol of H_2_ are theoretically produced for each mole of consumed glucose [4]. Theoretically, also in CLF, two moles of H_2_ are produced for each mol of acetic acid, according to the Equation (2).

Y_H2_/Y_AA_ = 2 (2)

On these bases, the ratio expressed in Equation (2) is about 2.0 and 1.6 for wt and *Acs03,* respectively. Hydrogen recovery was close to 100% in wt*,* and the experimental production (2.89 ± 0.39 mM) was very similar to expected theoretical amount (2.85 ± 0.1 mM) (Figure 3). On the other hand, in *Acs03* the H_2_ recovery was around 80% and the experimental production (1.92 ± 0.19 mM) was significantly lower than the theoretical value (2.37 ± 0.12 mM). Hydrogenase in *T. neapolitana* belongs to flavin-based electron bifurcating (FBEB) Fe-Fe hydrogenases and catalyses the oxidation/reduction of NADH and ferredoxin simultaneously in a 1:1 ratio to evolve H_2_ [36]. It utilizes the exergonic oxidation of Fd (*Em* = −453 mV) to drive the unfavorable oxidation of NADH (*E*_0_ = −320 mV) to produce H_2_ (*E*_0_ = −420 mV). The overall hydrogenase reaction can be described as follows:

NADH + 2Fd_red_ + 3H^+^ → 2H_2_ + NAD^+^ + 2Fd_ox_ (3)

Thus, loss in H_2_ yield with *Acs03* is very likely related to the increased consumption of NADH for the additional synthesis of lactic acid with consequent reduction of the availability of this cofactor for the H_2_ production. Under CLF, in addition to energetic flow derived from glycolysis, flavin-based enzymes NFN and RNF are suggested to be involved in the supply of reduced ferredoxin and NADH to support concomitant synthesis of lactic acid and hydrogen [17]. DNA perturbation imposed by heterologous expression of ACS probably impacted this electron balance circuit, lowering the general yield of hydrogen as electron sink. In theory, the demand of NADH might also be sustained by H_2_ consumption in recombinant strain.

A metabolic reorganization in microorganism overexpressing acetate activating pathway has been reported with conflicting results [37,38,39,40]. Zhang engineered *E. coli* with ACK-PTA and ACS pathways to increase N-acetylglutamate (NAG) production from acetate. The strain overexpressing ACK-PTA pathways increased NAG production 77.9% compared to the wild type strain, while the strain expressing ACS resulted in the biomass and NAG production about 40% lower than the wild type [40]. In contrast, the ACS pathway has been reported as the best choice for itaconic acid production in *E. coli*. In conditions tested by Noh [39], ACK-PTA pathway did not affect acetate consumption and itaconic acid production and caused a significative biomass reduction (wt, 0.81 ± 0.05 g/L; ACS expressing, 1.25 ± 0.12 g/L; ACK-PTA expressing, 0.21 ± 0.01 g/L) [39]. It worth noting that, in ACS-expressing systems, a significant effect is caused by higher ATP demand of this acetate activating pathway [41].

## 4. Conclusions

A metabolic engineering approach has boosted acetate uptake and reductive carboxylation of acetyl coenzyme of CLF pathway in *T. neapolitana* DSM33003. The enhancement was achieved by transformation with heterologous ACS-coding gene from *T. thermophilus*. Detailed characterization of fermentation products of the recombinant strain proved the restructuring of metabolic fluxes and resulted in a doubled production of lactic acid and a reduction of H_2_ production. Batch fermentations with exogenous ^13^C-labeled acetate established that the increased lactate in recombinant strain derived both from anabolic and catabolic branch of CLF. Forthcoming studies will focus on selective approaches of strain engineering to increase CO_2_ fixation into lactic acid without impairing hydrogen production.

## Figures and Tables

**Table 1:** Genetic manipulation reported for *Thermotoga* genus. Antibiotic (ant), auxotrophy (aux), and spheroplast (sph). All replicating plasmid used in the above-mentioned studies are based on the sequence of endogenous cryptic plasmid pRQ7.

Strain	Selection	Strategy	Transformation Technique	Target	Ref.
*T. maritima*	ant (kanamycin)	Replicating plasmid	sph-DOTAP/ electroporation	*kan* heterologous expression	[27]
	ant (kanamycin)	Replicating plasmid	sph-DOTAP	*kan* heterologous expression	[30]
	aux (uracil)	Chromosomal recombination	sph-DOTAP/ natural transformation	knock-out & knock-in *araA*	[34]
	aux (uracil)	Chromosomal recombination	natural transformation	knock-out *malk*X genes	[33]
	ant (kanamycin)	Chromosomal recombination	electroporation	transient inactivation of *ldh*	[33]
	aux (uracil)	Chromosomal recombination	electroporation	knock-in *malk3*	[33]
*T.* sp. RQ7	ant (kanamycin)	Replicating plasmid	sph-DOTAP/ electroporation	*kan* heterologous expression	[27]
	ant (kanamycin)	Replicating plasmid	natural transformation	*kan* heterologous expression	[29]
	aux (uracil)	Replicating plasmid	natural transformation	*pyrE* heterologous expression	[32]
*T.* sp. RQ2	ant (kanamycin)	Replicating plasmid	natural transformation	*kan + amya-celB* heterologous expression	[31]
	ant (kanamycin)	Replicating plasmid	natural transformation	*kan + xynb-celB* heterologous expression	[31]
	ant (kanamycin)	Replicating plasmid	natural transformation	*kan + xynb-celA* heterologous expression	[31]
*T. neapolitana*	ant (chloramphenicol)	Replicating plasmid	sph-DOTAP	*cat* heterologous expression	[30]

**Table 2:** Ratio of lactate (LA) and acetate (AA) yield in wild type (wt), empty vector, and ACS recombinant strain (*Acs03*).

	LA/AA
wt	0.44
Empty vector	0.36
*Acs03*	1.5

## Supplementary Materials

The following are available online at https://www.mdpi.com/article/10.3390/microorganisms9081688/s1.
