# Supplementary material for: Improvement of CO2 and Acetate Coupling into Lactic Acid by Genetic Manipulation of the Hyperthermophilic Bacterium Thermotoga neapolitana
Source: Microorganisms. 2021 Aug 9;9(8):1688. doi: 10.3390/microorganisms9081688 (PMC8399208; doi:10.3390/microorganisms9081688)
Supplement: Supplementary file 1 [file microorganisms-09-01688-s001.zip › microorganisms-1289645-supplementary.pdf]

Supplementary Materials

Thermotoga neapolitana [gbbct]: 68 CDS's (26795 codons)

fields: [triplet] [frequency: per thousand] ([number])

UUU 18.0(482)	UCU 11.0(296)	UAU 9.1(244)	UGU 4.8(129)
UUC 33.3(892)	UCC 13.5(362)	UAC 26.1(699)	UGC 3.2(86)
UUA 1.5(41)	UCA 7.1(189)	UAA 0.6(17)	UGA 1.8(49)
UUG 9.6(256)	UCG 7.9(212)	UAG 0.1(2)	UGG 15.1(405)
CUU 25.7(689)	CCU 10.1(270)	CAU 6.3(168)	CGU 3.0(81)
CUC 27.1(725)	CCC 11.6(310)	CAC 11.2(300)	CGC 2.1(57)
CUA 2.0(53)	CCA 10.4(280)	CAA 3.6(96)	CGA 2.9(78)
CUG 28.8(773)	CCG 10.2(272)	CAG 15.2(408)	CGG 2.5(66)
AUU 10.5(282)	ACU 5.6(150)	AAU 7.8(210)	AGU 7.6(204)
AUC 30.7(823)	ACC 12.6(337)	AAC 26.5(710)	AGC 7.4(199)
AUA 23.1(618)	ACA 13.2(355)	AAA 38.7(1036)	AGA 29.2(783)
AUG 22.3(597)	ACG 13.4(360)	AAG 35.5(951)	AGG 20.8(557)
GUU 23.9(640)	GCU 9.4(253)	GAU 29.1(780)	GGU 19.7(527)
GUC 16.8(449)	GCC 15.5(415)	GAC 25.9(693)	GGC 8.8(235)
GUA 8.9(238)	GCA 17.1(459)	GAA 52.9(1418)	GGA 34.8(933)
GUG 37.5(1005)	GCG 12.5(336)	GAG 37.9(1015)	GGG 9.0(240)

Coding GC 47.89% 1st letter GC 53.23% 2nd letter GC 35.40% 3rd letter GC 55.04%

Figure S1. Codon usage optimization from *T. neapolitana*. Data from http://www.kazusa.or.jp/codon/cgi-bin/showcodon.cgi?species=2337.

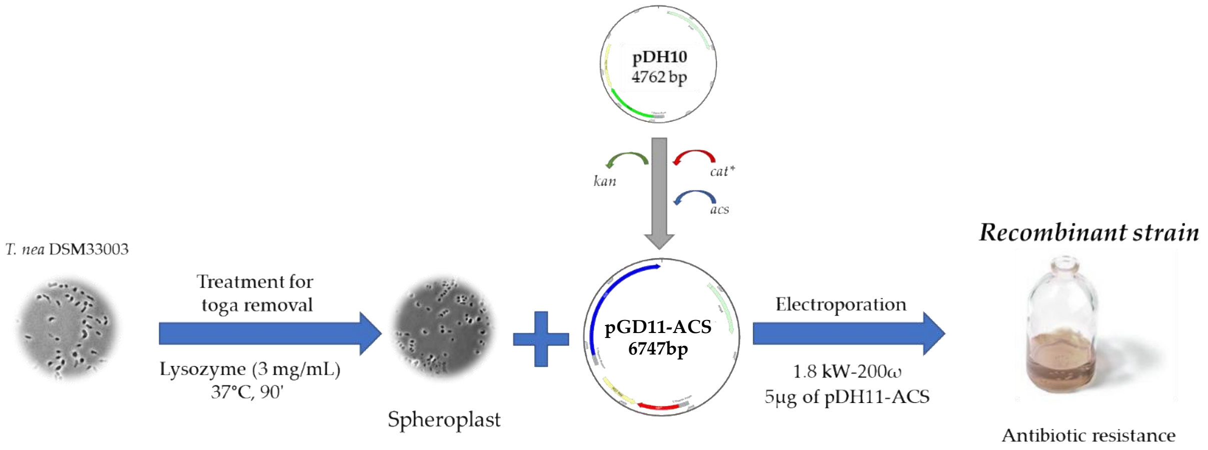

Figure S2. Transformation procedure optimized for *T. neapolitana* DSM33003

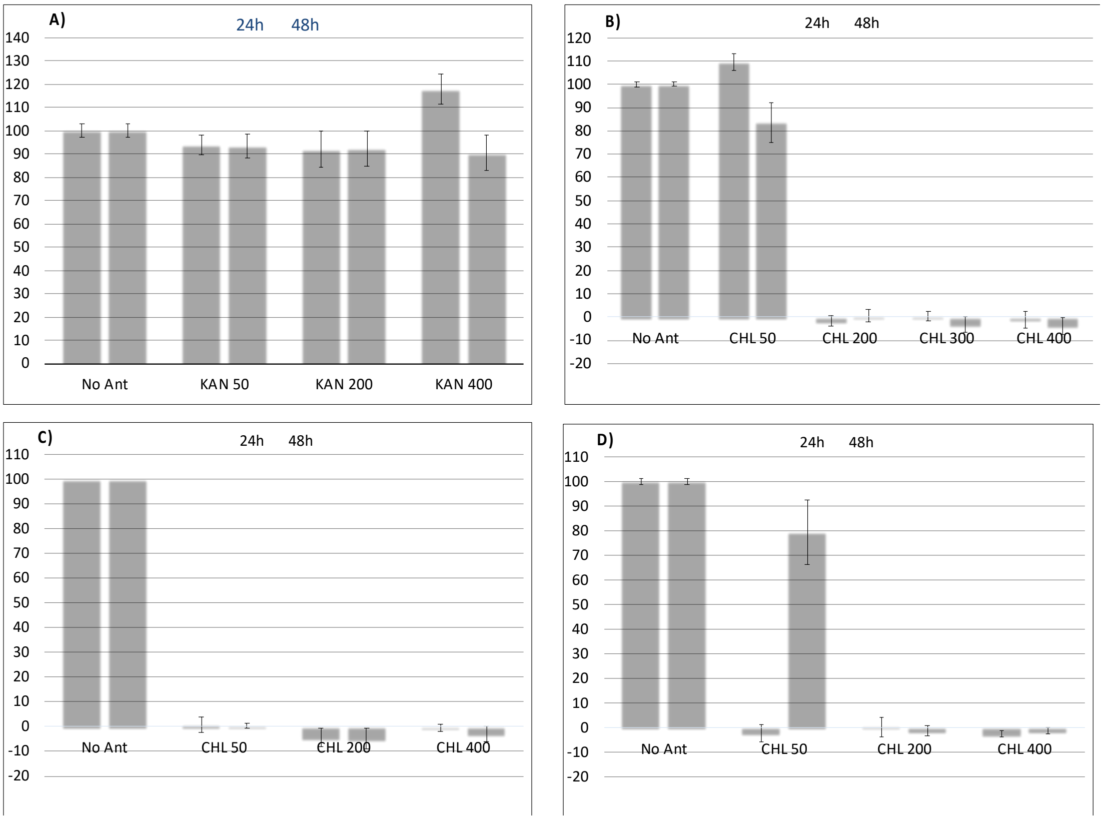

Figure S3. Sensitivity of *T. neapolitana* DSM33003 to kanamycin (KAN) and chloramphenicol (CHL) in liquid medium at optimal growing temperature (80 °C). Data are expressed as % of growth in comparison to control without antibiotic (No Ant, 100%). Kanamycin was tested at concentrations of 50 μg/mL (KAN 50), 200 μg/mL (KAN 200), and 400 μg/mL (KAN 400). Chloramphenicol (CHL) was tested at concentrations of 50 μg/mL (CHL 50), 200 μg/mL (CHL 200), 400 μg/mL (CHL 400). Data are expressed as mean of three replicates ± SD. A) and B): wild type; C) spheroplasts; and D) electroporated spheroplasts.

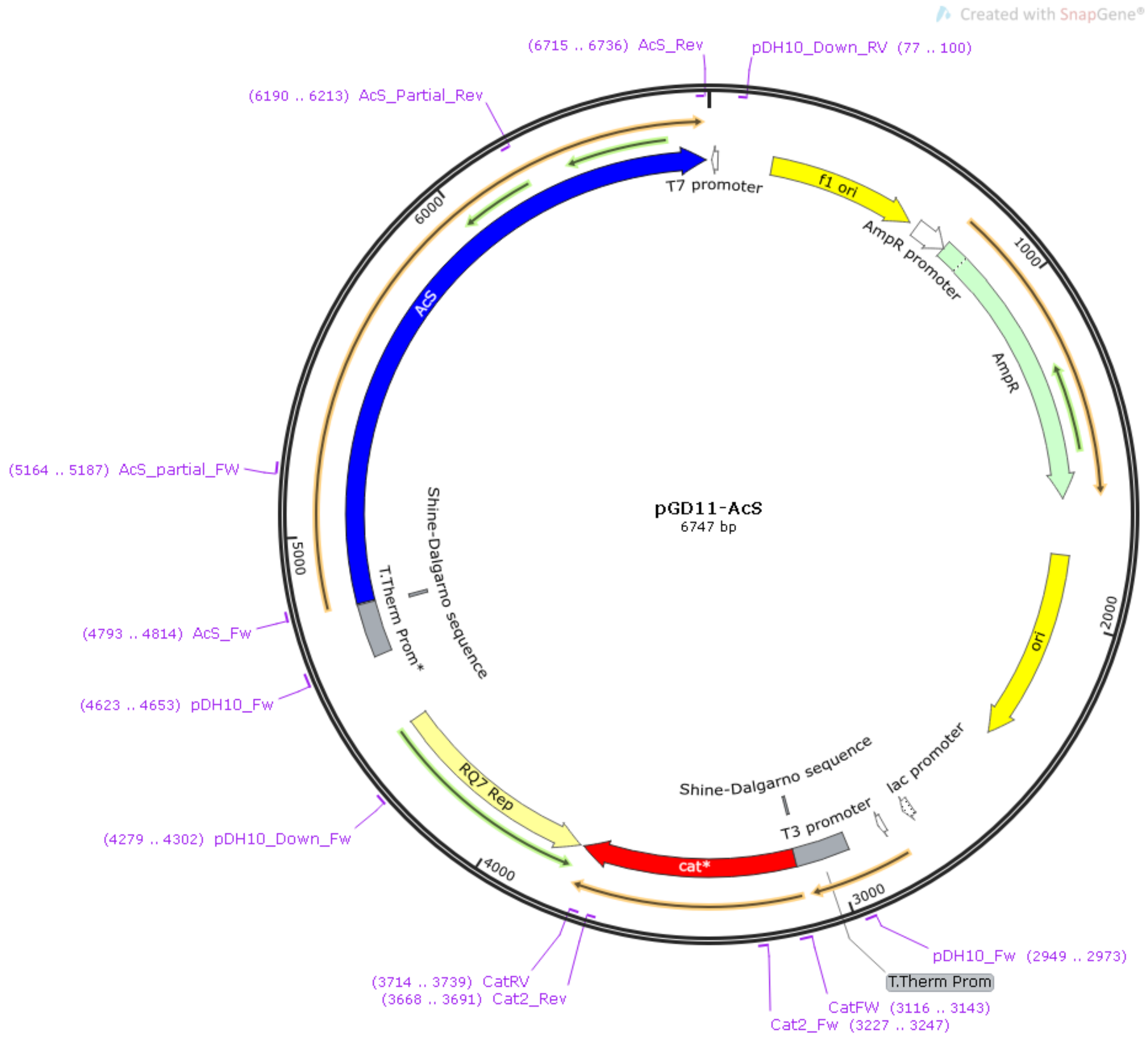

Primer	Sequence
acs_Partial_Fw	CGCCAACGTGCTGAAAAGACTGGG
acs_Partial_Rev	GGCCAAGGTCTCGTGATACACAGG
acs_Fw	ATGGATAGACTGGAATCCGTGC
acs_Rv	GGCTCCTTCTTTCAGTCTTTCC
pDH10_Fw	CTAGATTGACAAGGGCGCGTGAGGTTTTTA

Figure S4. Schematic representation of pGD11-ACS and primers used for ACS amplification (Table). Amp[r] is for amplification and selection in *E. coli*; Cat[r] is for selection in *T. neapolitana*; pRQ7 is for replication in *T. neapolitana*; and *acs* sequence and the selected promoter are from *T. thermophilus* HB8 (see manuscript for detail). For ACS detection, primer sequences are reported in the Table. Different combinations were tested: acs_Partial_Fw/Rv; acs_Fw/Rv; and pDH10_Fw/acs_Rv.

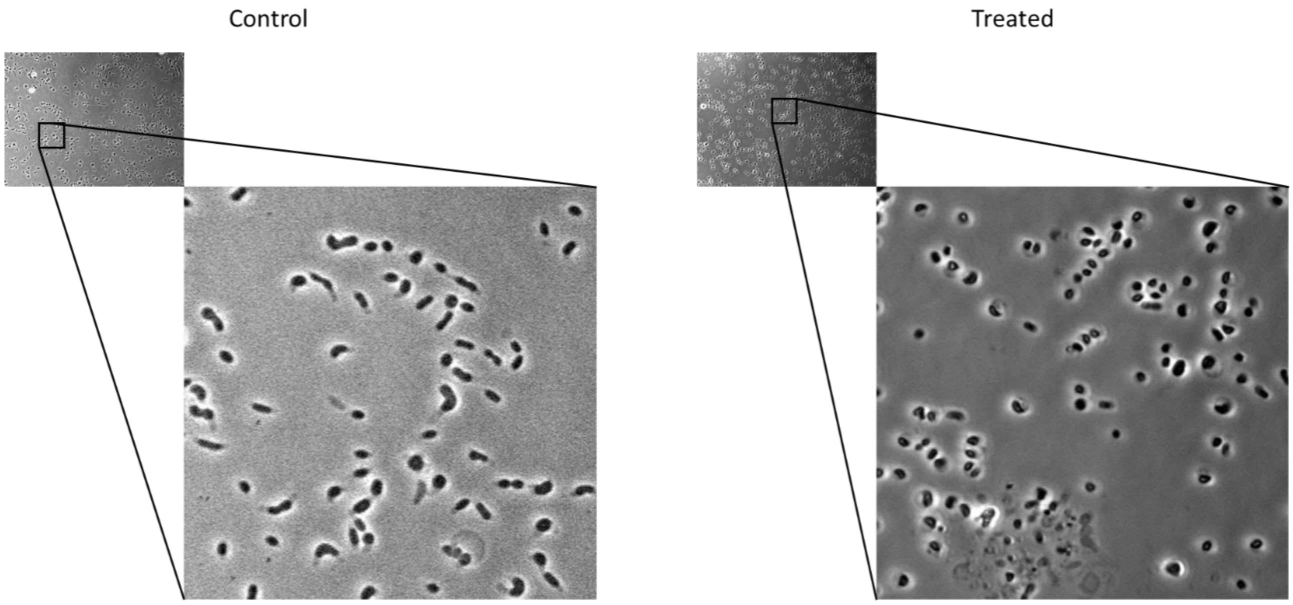

Figure S5. Spheroplasts of *T. neapolitana* DSM33003. Image before and after the treatment with lysozyme. Spheroplasts formation was monitored by a light microscope (Axio VertA1, Carl Zeiss, magnification of 100×).

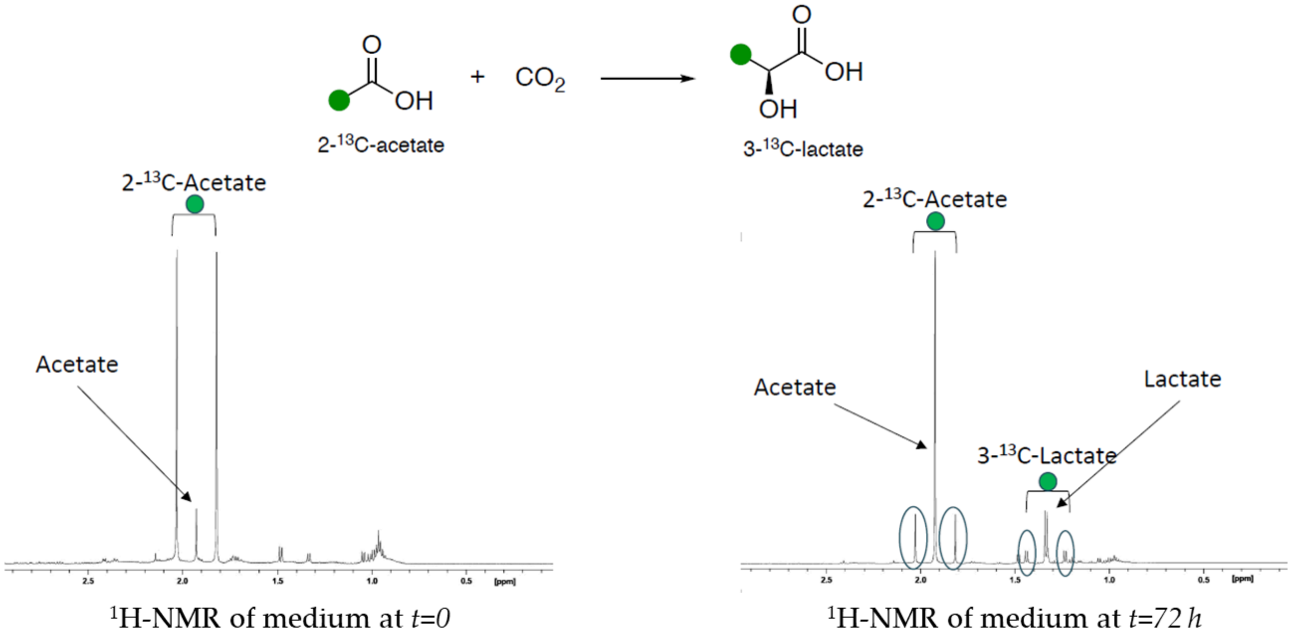

Figure S6. Scheme of CO_2 recycling and CLF reaction with 2-^{13}C labeled acetate. ^{1}H NMR spectra of the supernatants at t = 0 (left) and t = 72 h (right) of the wt strain.
